# The Impact of the COVID-19 Pandemic and Social Isolation on the Sexual Functioning of Women Who Have Been Treated for Vaginismus

**DOI:** 10.7759/cureus.28736

**Published:** 2022-09-03

**Authors:** Ebru E Zülfikaroglu

**Affiliations:** 1 Obstetrics and Gynaecology, Eva Women's Health Clinic, Ankara, TUR

**Keywords:** hdrs, griss, asex, cognitive and behavioral therapy, female sexual dysfunction, treatment outcome, vaginismus, covid-19 retro

## Abstract

Background: Multiple factors that impact the mental and hormonal condition of the person influence female sexual function. To our knowledge, however, hardly any research has investigated the sexual function during the COVID-19 pandemic for women who were treated for vaginismus.

Aim: The purpose of this research is to examine how sexual function, frequency of sexual activity, and dyspareunia altered in women who had been treated for vaginismus before the pandemic.

Methods: This observational study ultimately included 204 patients with completely treated vaginismus at the Women's Health Clinic . Before and during the pandemic, the following data was collected: age, education level, profession, frequency of sexual activity, Arizona Sexual Experiences Scale (ASEX) scores, Golombok-Rust Inventory of Sexual Satisfaction (GRISS) scores, and Hamilton Depression Rating Scale (HDRS) scores.

Results: Before the pandemic, the mean ASEX score of women in the research group was 12.56±3.41 , and during the pandemic, ASEX average scores of the women significantly increased to 16.88±5.56 . The GRISS total scores were 28.7±10.1 (range, 14-50) following therapy and 23.9±14.8 (8-58) during the pandemic. After therapy, the HDRS score was 9.58±5.53 (1-19) , while it was 15.21±6.43 (5-26) during the pandemic. During the coronavirus disease 2019 (COVID-19) pandemic, mental health declined significantly, indicating a mild state of depression unrelated to vaginismus. During the pandemic, the mean frequency of sexual activity was 2.9±1.4 per week, and the frequency of sexual encounters did not considerably vary.

Clinical Implications: Therapists should examine the pandemic's impacts on all sexual function symptom categories and modify their treatment plans appropriately. The findings indicate that lowering COVID-19-related stress may be especially useful in minimizing the negative impact of COVID-19 on symptoms. We noticed that vaginismus-treated women did not relapse during the pandemic.

Limitations: The study population was comprised of women attending a particular women health clinic. This can place a certain bias on the demography of the patient population.

Conclusion: The present research indicated that the frequency of sexual activity among women treated for vaginismus did not alter, and notwithstanding a rise in stress and depression ratings, the majority of sexual function scores, including pain, improved during the pandemic. Nonetheless, dissatisfaction and anorgasmia subscales deteriorated, while ASEX satisfaction did not improve to the same extent, suggesting deleterious consequences on sexual function.

## Introduction

Coronavirus disease 2019 (COVID-19), which was first seen at the end of 2019 in China, and then spread rapidly all over the world, had been declared a pandemic [1]. The COVID-19 pandemic caused massive societal, economical, and political turmoil on a worldwide scale and is widely considered the worst public health calamity of our time. People's mental health has been negatively impacted due to social isolation, sudden changes in everyday schedules, lengthy stays at home, and limits on socializing [2]. Common reactions to the global pandemic include worry for one's own or a loved one's health, as well as depressive or post-traumatic stress disorder symptoms [3,4]. Multiple factors that impact the mental and hormonal condition of the person influence female sexual function [5]. Negative effects on sexual function may be caused by factors including stress, changes in routine, and the fear of becoming unwell, since these factors can cause emotional and cognitive changes that divert attention away from sexual cues and have undesirable consequences on genital and subjective arousal [3,6]. The sexual health of individuals who are already facing more health problems globally could be affected by COVID-19.

The Diagnostic and Statistical Manual of Mental Disorders, Fifth Edition (DSM-5) classifies vaginismus as a subset of the genito-pelvic pain/penetration disorder in which any form of vaginal penetration, such as tampons, digits, vaginal dilators, gynecologic examinations, and intercourse, is frequently painful or impossible [7].

There have been studies investigating the impact of the COVID-19 pandemic on female sexual function [3,8]. To our knowledge, however, hardly any research has investigated the sexual function during the COVID-19 pandemic for women who were treated for vaginismus. The purpose of this research is to analyze the change in sexual function, frequency of sexual activity, and dyspareunia during the COVID-19 pandemic in women who have been treated for vaginismus.

## Materials and methods

A group of women who had been treated for vaginismus prior to the pandemic engaged in this observational research conducted in Eva Women's Health Clinic, Ankara, Turkey. A total of 204 subjects were included in the current study who did not meet the exclusion criteria that included: women who 1) were pregnant at the time of the research, 2) had vulvar vestibulitis syndrome, 3) had a history of tubo-ovarian abscess and pelvic inflammatory disease, 4) had diseases transferred by sexual contact, and/or 5) were hesitant to complete the questionnaires during a telephone interview. On the basis of the DSM-5 and the Structured Clinical Interview for DSM-5 Disorders (SCID-5) [7], a clinical psychologist made each and every diagnosis. In our study, which we published before COVID-19, all patients met the DSM-5 diagnostic criteria for vaginismus [9]. We were already asking these survey questions to all of our patients in order to assess the success of our therapy, and we were getting written agreement from them to participate in this survey. Also, since this research was conducted in accordance with the Helsinki Declaration, informed consent was obtained from all participants at the time of enrollment. When the COVID-19 pandemic started, the same questions were posed to the previously polled patients. Ethics committee permission was not required since it was a retrospective and observational research. The same professional gynecologist interviewed each participant individually.

Before COVID-19 broke out, we used sexual function questionnaires [9,10] to test a group of 512 patients with vaginismus. During the pandemic, we decided to recall and reexamine the same treated group. A reevaluation happened between March and April of 2022.The overall response rate was 39.8% (N = 204). During the pandemic, clinical factors investigated in the pre-COVID-19 research were re-evaluated, including Arizona Sexual Experiences Scale (ASEX), the female version of the Golombok-Rust Inventory of Sexual Satisfaction (GRISS) and Hamilton Depression Rating Scale (HDRS) that were previously administered. A total of 204 patients with vaginismus who had been asked to take part in the study did so. People filled out the questionnaires and took part in the study online, over the phone, or in person. From the women's medical records, demographic information and the assessment of female sexual function before to the pandemic were gathered. Three months following their first sexual encounter, each woman had got a standard gynecologic checkup. The frequency of sexual activity was recorded, and a visual analog scale was used to assess pain. The ASEX and the female version of the GRISS were used to assess sexual function, while the HDRS was used to assess depression. They reported having successful sexual encounters following therapy and during the three-month follow-up. No feedback was received indicating the recurrence of the problem.

The ASEX, developed by McGahuey et al. [11], was applied to evaluate the sexual activity of the couples. The Turkish version, of which validity and reliability studies were conducted by Soykan [12], was used. The score ranged between 5 and 30, and a higher total score indicated sexual dysfunction. In this study, scores ≥11 were considered the cut-off, as suggested by Soykan. The female version of the GRISS is a 28-item questionnaire used to measure heterosexual couples' sexual dysfunction [13]. It includes subscales for anorgasmia, vaginismus, non-communication, infrequency, female avoidance, female non-sensuality, and female unhappiness. The higher the score, the worse the sexual function, and subscale values > 5 suggest sexual dysfunction. GRISS has been certified in Turkish [14]. The HDRS is a 17-item self-report rating inventory that examines severity of depression attitudes and symptoms [15]. The range for the total score is 0 to 53. Between 0 and 7 there is no depression, 8 to 13 there is mild depression, 14 to 18 there is moderate depression, and 19 to 22 there is severe depression. The HDRS has been recognized in Turkish [16]. The ladies were given a specific space for completing the ASEX, GRISS, and HDRS using traditional pen and paper. All ladies independently completed the surveys. During the pandemic, telephone interviews were conducted to determine the existence and frequency of sexual activity, obstetric history, and preferred method of contraception, as well as to administer the ASEX, GRISS, and HDRS. The severity of dyspareunia was measured using a visual analog scale.

The most important results of the study were how the pandemic affected the ASEX, GRISS, and HDRS scores. At the time of the COVID-19 pandemic, the secondary effects were a change in how often people were sexually active and a change in how painful sexual activity was on a visual analogue scale.

For statistical analysis, IBM SPSS Statistics for Windows, Version 25.0 (Released 2017; IBM Corp., Armonk, New York, United States) was used. The data was presented using the mean standard deviation format. In addition, the homogeneity of variances, which is one of the prerequisites of parametric tests, was checked with the Levene test. Normality assumption was analyzed using the Shapiro-Wilk test. p<0.05 was considered to be statistically significant.

## Results

Demographic and clinical characteristics of participants

The 204 women in the research group had an average age of 28.8 ± 4.8 (range, 20-45). The most prevalent educational level was a bachelor's degree (50.49%). Few women reported active outside employment, and the vast majority described themselves as "housewives". The average length of marriage was 5.91 years (ranging from 21 months to 19 years), while roughly 9% of patients experienced symptoms for more than 10 years (Table 1).

**Table 1:** Demographic data of the study participants

Education Level	n (%)
Illiterate	2 (0.98%)
Under diploma	12 (5.88%)
Diploma	60 (29.41%)
Bachelor degree	103 (50.49%)
Master of Science	21 (10.29%)
Doctorate	6 (2.94%)
Occupation Status	
Employee	23 (11.27%)
Public servant	35 (17.15%)
Business	18 (8.82%)
Worker	20 (9.8%)
Retired	0
Housewives	108 (52.94%)
Marriage Duration	
1.5-5 years	144 (70.58%)
6-10 years	41 (20.09%)
> 10 years	19 (9.31%)

At the time of the research, none of the couples had contracted COVID-19 infection. According to feedback given 16 weeks after hospital discharge, they experienced no recurrence of vaginismus.

Differences between participants' scores before and during COVID-19

Only five (2.45%) of the 41 women (20.09%) with a history of depression or panic disorder were taking antidepressant medication. The women received contraception advice and filled out the ASEX, GRISS, and HDRS. The average number of sexual encounters per week was 3.1±1.4 , and the average visual analog scale for pain score was 5.8±2.6. Before the pandemic, the mean ASEX score of women in the research group was 12.56±3.41 , and during the pandemic, the ASEX average scores of the women significantly increased to 16.88±5.56 (p=0.001) (Table 2).

**Table 2:** Comparison of the ASEX scores of women before and during the COVID-19 pandemic ASEX: Arizona Sexual Experiences Scale; COVID-19: coronavirus disease 2019

	Before pandemic	During pandemic	p-value
ASEX	12.56±3.41	16.88±5.56	0.001

The distribution of ≥11 ASEX scores of women in the research group is shown in Table 3. Before the pandemic, 57.84% (n=118) of women in the research group had sexual disorders. During the pandemic, this ratio increased to 75.98% (n=136). 

**Table 3:** Comparison of the ASEX ≥11 scores of women before and during the COVID-19 pandemic ASEX: Arizona Sexual Experiences Scale; COVID-19: coronavirus disease 2019

	Before pandemic	During pandemic	p-value
ASEX	118 (57.84%)	136 (75.98%)	0.024

The GRISS total scores were 28.7±10.1 (14-50) following vaginismus therapy and 23.9±14.8 (8-58) during the pandemic. The overall score and the areas of infrequency, non-communication, avoidance, vaginismus, and non-sensuality improved significantly. However, ratings in the dissatisfaction and anorgasmia areas declined dramatically. Table 4 presents a summary of the outcomes. 

**Table 4:** Results of the GRISS post-treatment and during the COVID-19 pandemic GRISS: Golombok Rust Inventory of Sexual Satisfaction; COVID-19: coronavirus disease 2019

	Before pandemic	During pandemic	p-value
Infrequency	4.4±1.6	3.5±2.4	0.009
Non-communication	4.1±1.9	3.0±1.6	0.004
Non-sensuality	4.4±1.5	2.5±1.3	0.025
Avoidance	3.9±1.3	1.6±1.1	0.008
Dissatisfaction	3.9±2.4	5.7±2.4	0.001
Vaginismus	4.3±2.1	2.5±1.1	0.023
Anorgasmia	3.5±1.5	5.2±2.1	0.002
Overall score	28.7±10.1	23.9±14.8	0.04

After therapy, the HDRS score was 9.58±5.53 (1-19), while it was 15.21±6.43 (5-26) during the pandemic. During the COVID-19 pandemic, mental health declined significantly, indicating a mild state of depression unrelated to vaginismus. Table 5 presents a summary of the outcomes. 

**Table 5:** Results of the HDRS post-treatment and during the COVID-19 pandemic HDRS: Hamilton Depression Rating Scale; COVID-19: coronavirus disease 2019

	Before pandemic	During pandemic	p-value
HDRS	9.58±5.53	15.21±6.43	0.001

The average frequency of sexual activity was 2.9±1.4 per week during the pandemic, while the visual analog scale for pain was 2.5±1.8. The frequency of sexual encounters did not considerably vary. Nonetheless, pain scores decreased significantly (P = 0.001) during the pandemic.

## Discussion

This present study aimed to examine the female sexual function of a group of patients who had been treated for vaginismus prior to the COVID-19 pandemic, the results following vaginismus treatment, and during the pandemic period. In addition to this, the study intended to investigate changes in vaginismus symptom dimensions and overall severity based on stress reactions related with COVID-19. A woman's sexual desire and frequency of sexual intercourse are known to be affected by a stressful lifestyle. With high levels of chronic stress and following earthquakes, decreased sexual desire and frequency of sexual intercourse have been recorded [17]. In contrast, it has been shown that sexual activity among women rises during stressful periods [18]. We anticipated that the anxiety caused by COVID-19 would play a key role in explaining the shift in these symptoms that occurred as a direct result of the pandemic. We observed in this study that women treated for vaginismus did not have secondary vaginismus symptoms during the pandemic.

Following the announcement of restrictions in most nations impacted by the COVID-19 pandemic, studies on how sexual life was affected were published. There is a connection between sexual behavior and psychological well-being [19]. Reduced libido is a common side effect of stress [20]. Women, students, and those experiencing certain medical symptoms were shown to have a more severe psychological response to the COVID-19 pandemic in its early stages [21]. Contradictory findings exist about the impact of the pandemic on female sexual function. A research was done in Italy discovered a reduction in sexual function and quality of life in women during the COVID-19 pandemic's social restrictions era [3]. They showed that, compared to before the pandemic, the number of women having sexual intercourse four times a month fell from 89 to 52 [3]. In a research done in the United Kingdom, it was discovered that 60.1% of the 868 participants were not sexually active throughout the pandemic. According to their findings, being male, younger, married, and drinking alcohol were related with increased sexual activity [22]. Li et al. observed that both young men's and women's sexual activity and sexual satisfaction reduced at the height of the COVID-19 pandemic in China [23]. Yuksel and Ozgor looked at how the COVID-19 pandemic changed the way Turkish women behaved sexual acts [8]. During the pandemic, the authors discovered that the frequency of sexual intercourse rose dramatically, but the Female Sexual Function Index (FSFI) scores deteriorated. Furthermore, the pandemic may disrupt relationship dynamics, which may negatively impact marriage life and sexual performance [24].

According to the findings of our research, the average ASEX score for females rose from 13.47 to 17.01 points. However, the findings from the women who participated in our research show a rise in female sexual disorder (FSD) outcomes after COVID-19, which is comparable with the findings from other countries. Although depression ratings deteriorated throughout the pandemic, sexual activity did not change, and pain and overall ASEX levels increased. During the pandemic, the GRISS overall score increased, while the dissatisfaction and anorgasmia domains declined [25]. Women had higher HDRS scores during the pandemic, which might be related to the psychological repercussions of the pandemic, such as fear of catching the virus and anxiety over social isolation and lockdowns, according to our findings. The GRISS dissatisfaction and anorgasmia subscales indicate that the quality of intercourse reduced during the pandemic, despite the fact that the frequency of intercourse remained constant and the visual analogue scale for pain decreased.The fear of contracting a disease from a partner during sexual activity via kissing or other personal touch may impair sexual performance.

The existence of phobic responses among our patients may have impacted their melancholy and anxious symptoms in response to the pandemic [26]. Long-term mental symptoms associated with the pandemic, such as fear, concern, and discomfort, have been named "coronaphobia" and are more common in those who are anxious and have a history of mental health disorders [27]. Anxiety and sadness have been linked to increased pain severity and sexual problems in women with vaginismus, suggesting that a highly stressful situation, such as a lockdown, may result in a decrease in sexual function [27]. Both sexes are susceptible to orgasmic dysfunction and anorgasmia while under lockdown for long periods of time [27]. The rise in the GRISS discontent and anorgasmia subscales during the pandemic may potentially be connected to the shift in pair dynamics seen in our research.

Women receiving medical care for genito-pelvic pain/penetration problem during the pandemic would benefit from psychosocial support to help them relax, feel less anxious, and improve their relationships with their partners and their ability to have sexual relations with them. In many nations, sexual and reproductive health has been negatively impacted by changes in healthcare [28]. Important to the response to the pandemic are the availability and accessibility of high-quality services for women [29].

During the pandemic, none of the women in our study reported reoccurring vaginismus, and they continued to have pleasant penetration with a reduction in pain ratings; this may explain the improvement in GRISS scores despite the pandemic.

The currently available research is advantageous in a number of ways. First, there was a sufficient number of vaginismus patients employed as the sample group. Second, we were able to evaluate and contrast life quality and sexual function both before and during the pandemic. Thirdly, we made use of sexual and mental health criteria that have been shown to be reliable. Our study is constrained by the fact that not all of the women who were treated for vaginismus issues gave their informed agreement to participate; the women who were included in the study were questioned over the phone.

## Conclusions

The present research indicated that the frequency of sexual activity among women treated for vaginismus did not alter, and notwithstanding a rise in stress and depression ratings, the majority of sexual function scores, including pain, improved during the pandemic. Nonetheless, dissatisfaction and anorgasmia subscales deteriorated, while ASEX satisfaction did not improve to the same extent, suggesting deleterious consequences on sexual function. As a result, therapists should examine the pandemic's impacts on all sexual function symptom categories and modify their treatment plans appropriately. We noticed that vaginismus-treated women did not relapse during the pandemic. Future research might examine the impact of psychological assistance on these women's ability to deal with stress, anxiety, and depression, as well as their sexual function.
